# The Impact of Humidity in the Thermal Ageing of Celluloid: An Inter-Scale Investigation

**DOI:** 10.3390/polym17121648

**Published:** 2025-06-13

**Authors:** Anna Piccolo, Patrizia Tomasin, Alfonso Zoleo, Enrico Miorin, Luca Nodari

**Affiliations:** 1 Department of Chemical Sciences, University of Padova, Via Marzolo 1, 35131 Padua, Italy; alfonso.zoleo@unipd.it; 2 Institute of Condensed Matter Chemistry and Technologies for Energy, National Research Council (ICMATE-CNR), C.so Stati Uniti 4, 35127 Padua, Italy; patrizia.tomasin@cnr.it (P.T.); enrico.miorin@cnr.it (E.M.)

**Keywords:** celluloid, ageing, ATR-FTIR spectroscopy, Raman spectroscopy, electron microscopy, cultural heritage

## Abstract

The role of humidity on the ageing of celluloid is investigated by performing accelerated ageing tests on mock-up samples. At 70 °C, three levels of relative humidity (RH) are selected: 30%, 50%, and 70%. Samples are monitored for the macro- and micro- changes occurring through ageing to relate the visible modifications to the molecular ones. Infrared and Raman spectroscopy, microscopy, mass and contact angle measurements, profilometry, and colourimetry are combined for this purpose. While the ageing test at 30% RH results in a slight embrittlement of the samples and small spectral changes, the one at 50% RH induces significant modifications at the molecular level and the formation of cracks, while the one at 70% RH causes a fast deformation of the samples and the development of bubbles. Although quite diverse, such results prove to be related to the same chemical processes: denitration, chain scission, and oxidation. These occur more promptly or extensively based on humidity level, resulting in different outcomes. Beyond morphology and brittleness, macroscopic effects also involve mass loss, surface roughening, and yellowing. A possible correlation between the macro and micro modifications is present, highlighting the influence humidity has on the degradation process of celluloid.

## 1. Introduction

Celluloid can be considered to be the first successfully commercialised plastic [1]. Like all other synthetic materials that were produced later, very little is known about its durability, given that less than two hundred years have passed since its introduction. Yet, signs of degradation have already been observed and give rise to the necessity of investigating its alteration behaviour.

Celluloid is the commercial name of a physical blend of cellulose nitrate (CN) and camphor, the latter acting as plasticiser. Such flexible thermoplastic material was introduced in the 1870s, after the debut at the Great International Exhibition (1862) of cellulose nitrate, and was widely employed for about a century [1,2,3,4,5]. Depending on the other additives included in its production, celluloid served as a cheaper and easily accessible substitute for luxury materials, such as ivory and tortoiseshell, and was utilised to produce billiard balls, dentures, and households, as well as X-ray, photographic, and cinematographic films and animated cells [6,7,8,9,10]. Moreover, artists like Antoine Pevsner, Naum Gabo, and László Moholy-Nagy used celluloid as a medium for some of their creations [1,2,11,12]. Due to the unstable nature and flammability of CN, its use was progressively abandoned in the first half of the 20th century in favour of safer formulations [13,14]. Still, this material composes a number of testimonies to the start of the “plastic era”—among which are design items, artworks, historical objects, and images—that often present non-negligible signs of degradation and, therefore, risk being lost.

Studies and debates started to focus on plastic degradation in the 1990s, when it became evident how such recent materials were prone to fast ageing processes and, therefore, deserved particular attention for their conservation [1,13]. The first challenge is usually material identification, since plastic objects are generally imprecisely catalogued and their appearance can be misleading [10,14]. The study of celluloid degradation phenomena also raises several questions. The mechanisms are generally agreed to comprise a denitration process, which leads to a decrease in the degree of substitution (DS) and, by releasing nitrogen oxides, renders the alteration process autocatalytic and hazardous to nearby objects [2,13,15,16]. Oxidation products with lactone and anhydride functions can be produced and hydrolysis reactions can be favoured [17,18,19]. Both the processes lead to chain scission and, therefore, to a loss of molecular weight, which is the main cause for the physical modifications of CN objects [17,18].

During recent decades, changes in the macro-properties have been investigated by different authors through techniques such as micro-hardness tests, gloss measurements, and colourimetry [20], whereas the corresponding modifications at the molecular level have been analysed using ion chromatography, UV-VIS luminescence, and infrared and Raman spectroscopy [6,12,16,17,18,19,21]. Albeit widely studied, the degradation mechanism of celluloid is still a matter of debate, and further research is needed for a deeper and more comprehensive understanding of its unfolding. To this end, the use of mock-up samples is pivotal, as it allows for detailed investigations using techniques that could not be employed on cultural heritage objects due to their invasiveness. The identified markers for the occurrence of degradation can be then at the basis of a non-invasive diagnostic campaign on museum objects. The alteration can be triggered on mock-up samples through accelerated ageing experiments, which, despite involving environmental conditions that are significantly different from the real ones, give the means for a better understanding of the material’s behaviour in a research-convenient timeframe. The accelerated ageing protocols proposed in the literature for the study of celluloid are, up to now, quite diverse in nature [2,16,17,19,20,21,22,23,24]. Given the need for clarity, the authors aim at filling in these research gaps by offering a reproducible procedure in which the main environmental factors, i.e., temperature (T), relative humidity (RH), and light exposure, are systematically varied. For the present work, ageing experiments were performed in a dark environment to neglect the effect of light; the temperature was set to 70° C to speed up the degradation phenomena and for the sake of comparability with previous studies carried out at this temperature [2,19,21]. Three levels of humidity were considered to investigate its role in the alteration of celluloid at 70 °C. Ultimately, this study aims to set the basis for proposing the optimal conditions for the preservation of this material. Both macroscopic and chemical modifications are considered in order to provide an extensive outlook on the ageing of celluloid and highlight the significant parallelisms between the visible changes in the material and its molecular alteration.

## 2. Materials and Methods

### 2.1. Choice of the Samples and Sample Preparation

Commercial celluloid films were studied as replicas of the historical material, which was of industrial quality. Given its high flammability, celluloid is now scarcely retrievable from the market. Transparent and uncoloured (200 × 100 × 0.5) mm^3^ sheets were purchased from Rothko&Frost [25], which still sells celluloid; the lowest available thickness was preferred to have a material like that commonly used for films, and both colour and opacity were avoided so as to study the simplest system possible, made of only cellulose nitrate and camphor. The purchased sheets of celluloid were partitioned into smaller pieces, with the aim of having suitable samples for the different experiments and analyses. Scissors were employed to cut the samples into squares of 1, 2, or 3 cm edges, whereas a scalpel was used to mark the side of the sample facing the holder.

### 2.2. Accelerated Ageing

In this work, a T of 70 °C was chosen for the accelerated ageing experiments to foster the degradation processes and to be sure to observe significant changes, while the RH% was varied to have an outlook on its role. Samples were placed on Petri dishes inside glass desiccators. Such systems were positioned into an oven, and their relative humidity was regulated by means of targeted saturated salt solutions. Sodium chloride, magnesium nitrate, and magnesium chloride were chosen based on the work by L. Greenspan [26] to attain levels of 70, 50, and 30% RH, respectively. Ageing systems were stabilised overnight before starting the tests, and sampling was performed at most once a day to minimise the perturbations to the environment. Such a protocol was based on a methodological study, which is briefly described in the supporting information (Supplementary Materials, Section 1). The three experiments had different durations based on their effect on the samples, which will be discussed in Section 3.1. Since such degradation effects precluded the performance of the routine analyses, the experiment at 50% RH was interrupted after 41 days and the one at 70% RH after one week. The ageing test at 30% RH was run for 48 days without reaching a critical stage of deterioration. It must be noted that some measurements could not be performed on the most aged samples due to their fragility.

### 2.3. Analytical Techniques

Multiple analytical techniques were employed to characterise the samples and monitor their changes throughout the ageing process. These served the purpose of investigating both the chemical and physical properties of the material, with the final aim of correlating the alterations occurring at the molecular scale to the visible macroscopic modifications.

The mass change was monitored using a Sartorius M2P micro balance. Repeated measurements were performed on three (1 × 1) cm^2^ samples in each ageing experiment at different time intervals.

The wettability of the material surface was evaluated by measuring the static contact angle. Drops of deionised water of about 10 µL were allowed to fall on a dedicated sample, and micrographs were acquired with a Dino-Lite digital microscope 0 s, 5 s, and 20 s after the deposition. The drop-analysis LB-ADSA plug-in of ImageJ 1.54g software was used to measure the contact angle. Five drops per analysis were measured, and mean values were considered for data evaluation.

The morphology was documented by means of a Dino-Lite digital microscope, and a Scanning Electron Microscope (SEM) was employed for characterising the surface at the micro-scale. SEM analyses were performed on samples coated either with graphite or gold with a FEI Quanta 200 FEG-ESEM instrument. For images acquisition, a high vacuum and a voltage of 1 kV were used, the spot size was set to 3.5, and the working distance was ca. 7.9 mm.

Further information on the surface morphology was obtained through profilometry. By using a Bruker Dektak XT stylus profilometer, multiple 4800 µm long scans were performed on each sample. Scans were acquired parallel either to the sides of the squared celluloid sheets or to their diagonals. In the former case, samples were scanned in both directions to avoid being influenced by the possible surface features produced by the extrusion process, which was the method used to produce the studied celluloid sheets. For the selected samples, the variation in the surface shape was evaluated through the calculation of two different quantitative parameters—the arithmetic mean roughness (Ra), determined by the roughness profile; and the arithmetic mean waviness (Wa), determined by the waviness profile—according to the indications of the ISO 21920-2 standard [27]. Moreover, 3D mapping was also acquired to obtain a better visualisation of the changes in the surface morphology. Maps were collected over areas of 800 µm × 800 µm.

A CM-700d spectrophotometer by Konika Minolta with a 6 mm diameter probe was employed for colourimetric analysis. Given the transparency of the studied material, samples were placed onto white paper sheets for the measurements, and the changes rather than the absolute values of L*, a*, and b* were evaluated. Five measurements were performed on different areas of each sample, and the mean values were considered in data handling.

Infrared spectra were recorded in attenuated total reflectance (ATR) and transmission mode. ATR-FTIR spectroscopy was employed for the routine monitoring of sample ageing with the use of a Bruker Alpha II instrument. For analysing samples sections, a LUMOS II µ-FTIR spectrometer in µ-ATR mode was used. In both cases, analyses were performed with repeated measurements by recording spectra in the range 4000–350 cm^−1^, with 256 scans and a resolution of 4 cm^−1^. For more detailed evaluations on the ageing trends, intensity ratios of the signals cantered at ca. 3430, 1728, 1635, 1160, and 1045 cm^−1^ ascribed, respectively, to ν O-H, ν C=O, ν_as_ O-N-O_2_, and ν C-O-C (the last two) were calculated after baseline correction. The carbon–oxygen stretching was chosen as the reference to be used for monitoring the change in nitrate groups in the material. Taking the works by Quye et al. [21] and Chavez Lozano et al. [19] as models, both the signals at 1160 and 1054 cm^−1^ were considered and were referred to as C1 and C2, respectively.

For transmission FTIR spectra, samples were either reduced into thin slices with the use of a scalpel or were grounded in a mortar, based on their elasticity. The former method was employed for pristine celluloid and mildly aged samples, whereas the latter was used for the embrittled aged material. Samples were mixed with potassium bromide at a mass ratio of 1:100 for preparing KBr pellets and were analysed with a Nicolet Continuµm spectrometer in the range 4000–400 cm^−1^. With the aim of comparing the surface and bulk chemical changes, such a technique was employed for the analysis of the pristine material and of samples aged for the maximum duration of the different thermal ageing experiments at 70 °C: 48 days at 30% RH, 41 days at 50% RH, and 7 days at 70% RH.

For complementary information, a Renishaw inVia Raman instrument equipped with a Leica confocal microscope system was utilised, employing a 633 nm laser. Spectra were acquired in the range 4000–100 cm^−1^, with the laser power set to 50%, with 50× magnification and 10 s of exposure. For data analysis, the baseline of the spectra was subtracted with Origin.

## 3. Results

### 3.1. Physical and Morphological Changes

**Figure 1:**
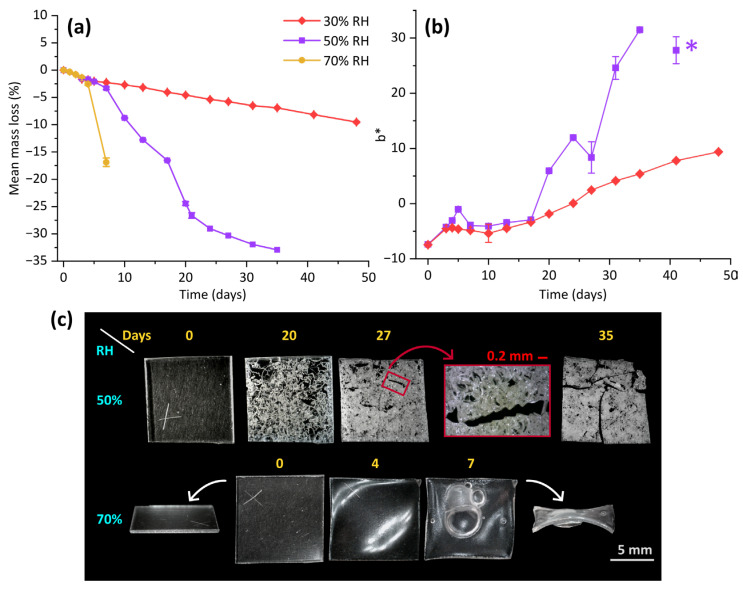
For celluloid samples aged at 70 °C and 30%, 50%, and 70% RH, the percentage mass loss (**a**) and b* colourimetric values (**b**) are plotted versus the time of ageing. No colourimetric measurements were performed for the ageing at 70% RH. In (**b**), the b* value collected on the 41st day of 50% RH ageing is indicated with a star (*), since it refers to the sample used for contact angle measurements. In (**c**), optical micrographs of the most significant morphological changes are displayed. The days of ageing are reported in dark yellow, while the RH% used for the ageing is written in light blue. For the 50% RH ageing, a detail of the cracks is shown for the 27th day, while for the ageing at a higher humidity, samples of the unaged material and that aged for one week are also shown on the edge.

Celluloid samples subjected to the thermal ageing experiments at 70 °C underwent significant changes in their macro-properties, which were more evident when ageing at higher humidity levels. A progressive mass loss was registered for all the experiments. While in the first three days they followed the same trend, the effect of moisture became evident subsequently (Figure 1a). For samples aged at 70% RH, a significant drop was registered after four days of ageing. Those subjected to 50% RH lost about one third of their weight after 35 days. Such a decrease followed a quite irregular trend, whose steepest part was between 7 and 20 days. At 30% RH, the loss of mass was rather linear, with a constant slope. After 48 days of ageing, less than 10% of weight was lost, not even reaching the result of a one-week ageing experiment at 70% RH.

Throughout the high-temperature ageing, samples underwent other macroscopic modifications. In the case of the high humidity conditions, a significant morphological modification was observed: samples started warping after 4 days, and in one week, they were distorted by several bubbles of varying dimensions and became quite brittle. For those aged at 50% RH, no change was detected for 17 days, whereas they appeared visibly damaged starting from the 20th day of ageing (Figure 1c). Cracks arose and spread with the passing of time, creating complex and interwoven patterns. The shape of such fissures was quite uneven, but generally was characterised by being wavy, as can be seen in the detailed view reported in Figure 1c. The fragility of the material increased considerably, so that safe handling became extremely problematic. Samples collapsed under the mere pressure exerted by the tweezers used for their manipulation. In the case of the 30% RH ageing, no visible morphological change was noticed, but celluloid sheets became more brittle, particularly after the 27th day. The brittleness they gained was far lower than that of the cracked samples, but was sufficient for breakage by the pressure of the ATR-FTIR instrument. Contact angle measurements showed a slight increase in hydrophobicity for these samples. Instead, for samples aged at higher humidities, wettability increased concurrently with crack formation in one case and bubble formation in the other (see Figure S2).

Colourimetric measurements enabled the recording of the yellowing process affecting the samples. This process followed an irregular trend that differed based on the level of humidity. It should be noted that the ageing at 50% RH ended on its 41st day; given that the sample dedicated to colourimetry was too damaged, L*a*b* data was collected on the piece of celluloid sheet employed for contact angle analysis. This measurement was aimed at documenting the dark hue gained by such sample, which ought to be considered separately from the rest of the colour change assessment. For the other samples, a slight shift toward greener hues was attested by the decrease in a* (see Figure S3), while L* values spanned in a range of about three units (the last point of the 50% RH ageing excluded), which was considered not remarkable. Contrarily, the increase in b* was noteworthy, especially for the samples aged at 50% RH (Figure 1b). Such a yellowing process was characterised by a small peak in the fifth day of ageing, followed by rather constant values until the seventeenth day and, subsequently, by a more pronounced change. At 30% RH, a relatively fast variation was observed in the first three days (equivalent to that at 50% RH), while the following changes became slower.

**Figure 2:**
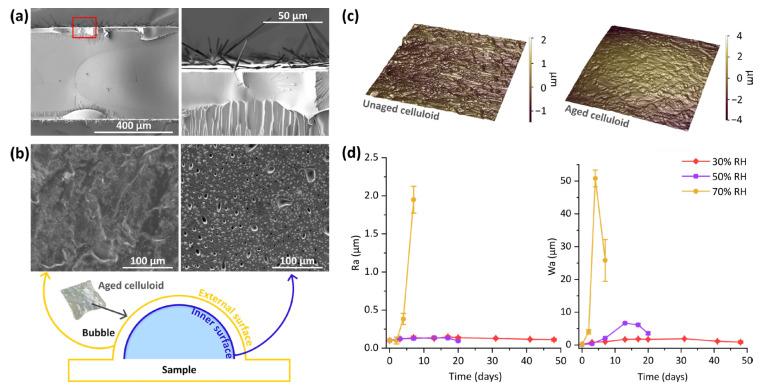
(**a**) SEM micrographs acquired on the section of a sample aged at 70 °C and 50% RH for 27 days (lower magnification on the left and zoomed detail of the red framed area on the right); (**b**) schematic of the bubbles formed upon ageing at 70 °C and 70% RH for 7 days and SEM micrographs of the inner and external surfaces; (**c**) profilometry maps acquired on the unaged material and on celluloid aged at 70 °C and 50% RH for 13 days; (**d**) plots of arithmetic mean roughness (Ra) and arithmetic mean waviness (Wa) values registered after different time intervals for samples subjected to the ageing experiments at 70 °C and 30%, 50%, and 70% RH.

SEM investigations revealed that 3 days of ageing were sufficient for the surface morphology to become less homogeneous, regardless of the level of humidity. Samples aged at 30% RH did not present visible changes throughout the rest of the ageing process. Crinkles of different orders of magnitude formed so that a wrinkled surface could also be affected by larger-scale deformations (Figures S4 and S5). The 50% RH thermal treatment led to the appearance of needle-shaped structures on the surface, starting from the 20th day of ageing. These were grouped in flakes protruding from the surface (Figure 2a) and were sometimes detected together with polygonal-like units. The latter were probably smaller elements of the same kind, as the needles’ section was found to vary among triangular, rectangular, and hexagonal shapes. Such structures were not observed in freshly cut sections of the samples, which were characterised by the presence of small hollows and cracks (for detailed micrographs on the samples with crystals, see Figure S6).

For the thermal ageing test at 70 °C and 70% RH, SEM observations were dedicated mainly to the samples aged for 7 days, which presented the most significant changes in roughness. The surface appeared rather inhomogeneous and marked by bubbles and pits. The sides of the fragments sampled for SEM investigations resulted in being broken into heterogeneous layers, presenting small holes of different shapes in the indentations (Figure S6). The bubbles formed upon ageing were also investigated by comparing the external and the internal surfaces, the former exposed to the surrounding environment and the latter facing the inside of the bubble. As can be seen by observing Figure 2b, the two surfaces were quite different—the internal one being characterised by a high number of fissures, with various dimensions and rather big blisters.

Changes in surface morphology were also investigated by means of profilometry through mappings and profiles. As can be seen by observing Figure 2c, the scale of surface roughness features increased with time of ageing. Modifications in both roughness and waviness were particularly pronounced for the samples aged one week at 70 °C and 70% RH (Figure 2d). Not only did the roughness of the surface rise quickly up to about 2 µm, but the distortion of the samples was quite remarkable. The high values of Wa well express the distortion of the samples, and the formation of bubbles occurred with the thermal ageing. For the other two levels of humidity, Ra values remained below 0.15 µm. The ageing at 30% RH did not cause noteworthy modifications in waviness, while the one at 50% RH resulted in a gradual rise, up to about 7 µm on the 13th day of ageing, followed by a drop to ca. 3 µm. Samples aged at 50% RH for more than 20 days were not analysed due to the extensive presence of cracks and to their extreme fragility.

### 3.2. Chemical Modifications

Infrared and Raman spectroscopy were employed for the assessment of the changes occurring at the chemical scale. The characteristic signals for celluloid were recognised in both cases (see Table S1 for complete band attributions).

ATR-FTIR spectra present a signal for the stretching of the hydroxyl group at ca. 3430 cm^−1^, C-H vibrations between 2970 and 2850 cm^−1^, the carbonyl band ascribed to camphor at 1728 cm^−1^, signals related to the stretching modes of N-O at 1635, 1273, and 828 cm^−1^, and those attributed to C-O stretching of the cellulose rings in the range 1160–990 cm^−1^ [17,18,19,28,29,30,31,32,33,34].

Raman spectra are characterised by the OH stretching at 3530 cm^−1^, C-H vibrations in the range 2970–2920 cm^−1^, the carbonyl band at 1730 cm^−1^, and NO-related signals at 1657, 1285, and 854 cm^−1^. In addition to the C=O stretching band, camphor gives rise to the signals at 1450, 863, 650, and 557 cm^−1^, the one at 650 cm^−1^ being given by the ring deformation [6,17,35]. The signals at 1417, 1373, and 1326 cm^−1^ can be ascribed to the scissoring of C-OH, that of the C-H bond, and to the wagging of CH_2_, respectively, while the bands in the range 1200–1000 cm^−1^ are associated with the carbon–oxygen vibrations of the pyranose rings and glycosidic bonds [6,17,35,36,37]. Further signals ascribed to the stretching N-OH, C-H scissoring, NO_2_ wagging, and O-N-O bending are centred, respectively, at 950, 915, 761, and 711 cm^−1^ [6,17,36].

The changes observed through the ageing experiments are hereafter presented by distinguishing the investigations performed at the surface (ATR-FTIR and µ-Raman spectroscopy) and in the bulk material (µ-ATR-FTIR, transmission FTIR, and µ-Raman spectroscopy).

#### 3.2.1. Changes at the Surface

**Figure 3:**
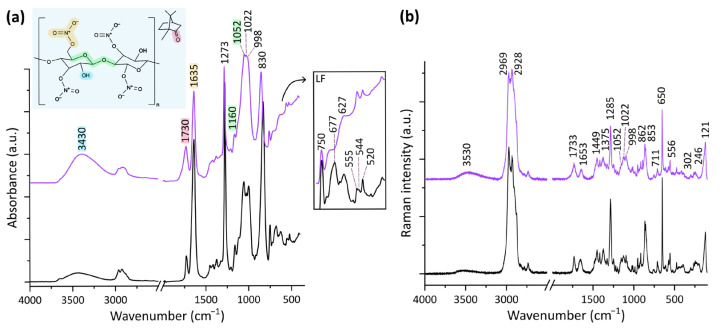
ATR-FTIR (**a**) and µ-Raman (**b**) spectra collected on the pristine material (black) and on samples aged for 41 days at 70 °C and 50% RH (purple). The wavenumbers of the discussed signals are reported in black. For the ATR-FTIR spectra, the signals considered for intensity ratios are coloured according to the corresponding functional groups highlighted in the chemical structure of cellulose nitrate and camphor. Moreover, a detailed view of the low-frequency region is shown in a box.

Significant changes were observed to occur throughout the thermal ageing by means of ATR-FTIR spectroscopy, particularly in the experiment where the humidity was set to 70% and 50%. These consisted of modifications in the relative intensity of the signals attributed to ν O-H, ν C=O and ν N-O, and ν C-O-C, as can be seen from the representative spectra reported in Figure 3. In particular, the increase in the absorbance for the hydroxyl group and the decrease in the intensity of bands attributed to the nitrate stretching modes were significant. A noteworthy broadening was observed for the carbonyl band, whereas the ν C-O-C signals in the range 1160–990 cm^−1^ changed in relative intensity.

Substantial changes were detected in the low-frequency range (760–400 cm^−1^) for the 50% and the 70% RH ageing tests, starting from the tenth and seventh day, respectively. The composite band, with maxima at 695 and 677 cm^−1^ attributed to N-O vibrations [30,38], decreased visibly in intensity, so that, after 40 days at 50% RH, it could be no longer distinguished. Similarly, N-O-related bands at 750, 650, 626, and 543 cm^−1^ and in the range 450–420 cm^−1^ gradually decreased in intensity (see inset in Figure 3a) [30,38]. Mukhamadeeva et al. [38] also reported that the two signals at ca. 553 and 522 cm^−1^ depend on the degree of substitution of the material. In particular, the signal at a higher frequency is said to gain intensity when the DS decreases. This is the case for the spectra collected for the samples aged at 50% RH: after 10 days, the absorbance of the band at 555 cm^−1^ was comparable to that of its neighbouring signal, until becoming the most intense of the two after 41 days. It should be noted that, also, camphor gives rise to signals at 553 and 522 cm^−1^, but this attribution alone does not explain the modification in relative intensity [19].

Raman spectroscopy provided further information on the chemical changes occurring in the samples. C-H stretching modes could be better characterised, so that a gradual change in the relative intensity of the signals at 2965 and 2930 cm^−1^ was observed. While the unaged material presented a stronger contribution of the first, the situation reversed after 20 days of ageing at 50% RH and 7 days at 70% RH. A broadening of the carbonyl band could be detected, as well as a decrease in intensity of the νas NO_2_ signal at 1653 cm^−1^ and an increase in the hydroxyl signal at about 3530 cm^−1^. The relative intensity of the C-O signals at 1151, 1121, and 1095 cm^−1^ changed through ageing, with a significant decrease in the first and a smaller, but not negligible, decrease in the second maximum. A decrease in intensity was also registered for camphor’s ring deformation band at 650 cm^−1^ in the case of the 50% RH ageing. Finally, the composite signal centred at 246 cm^−1^ underwent significant modifications after the third day of ageing, becoming narrower and gradually less intense.

#### 3.2.2. Changes in the Bulk Material

Spectral changes observed through transmittance FTIR for unaged and artificially aged celluloid reveal that transformations also occurred in the bulk of the material. These were very similar to the modifications assessed using ATR-FTIR spectroscopy, namely the changes in relative intensity of the signals given by O-H, C-H, C=O, and N-O stretching modes and the broadening of the carbonyl band, but differed partially (Figure S7). However, for the ageing at 30% and 50% RH, the band at 1735 cm^−1^ only grew broader with respect to the unaged sample; with 7 days of thermal treatment at 70% RH, a shoulder at 1780 cm^−1^ was evident. At lower wavenumbers, the signal at 1160 cm^−1^ became less resolved after 41 days of ageing at 50% RH and reduced to a shoulder of the neighbouring band for the higher-humidity scenario. The signals in the range 1070–990 cm^−1^ resulted in being quite different from those observed using ATR-FTIR spectroscopy, in that maxima were detected at ca. 1065, 1024, and 1005 cm^−1^ for the pristine material. The same situation was observed after the 30% RH ageing, while the bands evolved differently at higher humidity levels. For the 50% RH ageing, two maxima were registered at 1065 and 1054 cm^−1^ and three components could be distinguished at 1071, 1051, and 1024 cm^−1^ for the 70% RH ageing. For the experiments at medium and high levels of humidity, the low-frequency range of the spectra presented modifications as well, consisting of a decrease in intensity of the signals in the range 720–620 cm^−1^.

**Figure 4:**
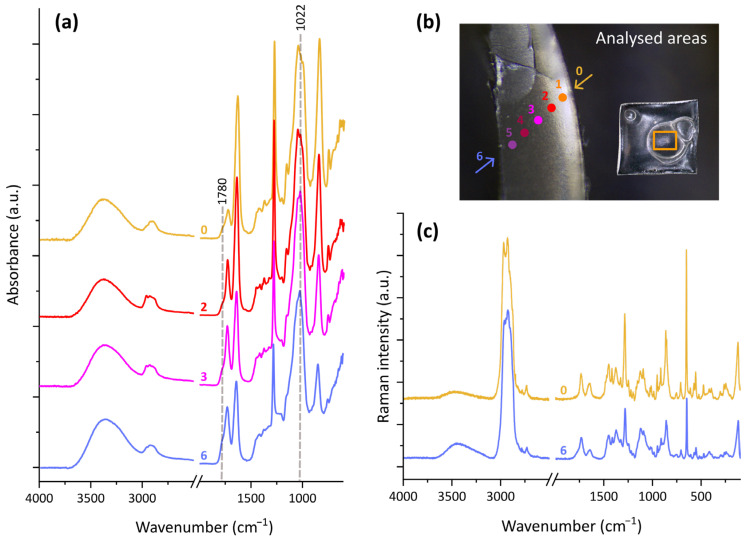
µ-ATR-FTIR (**a**) and µ-Raman (**c**) representative spectra collected through the section of bubbles formed on celluloid after 7 days of ageing at 70 °C and 70% RH. The approximate position of the analyses on the bubble section is shown in (**b**), with colours and numbers corresponding to those of the related spectra.

Changes in the relative intensity of the signals were also noticed when considering different depths across the upper part of the bubbles formed after 7 days at 70 °C and 70% RH. As can be seen in Figure 4a, the more one approaches the inside of the bubble, the more the afore-mentioned spectral modifications are evident in the IR spectra. These comprise the increase in intensity of the hydroxyl band, the weakening of the NO-related signals, and modifications of the bands attributed to carbonyl groups and C-O bonds, in the regions at 1800–1700 and 1170–900 cm^−1^ respectively. The signal centred at ca. 1730 cm^−1^ ascribed to the carbonyl group resulted slightly more intense and visibly broadened towards the inside of the bubble. Moreover, a shoulder at 1780 cm^−1^ turned out to be more pronounced at higher depths. Signals related to the C-O stretching vibrations visibly differed based on the depth of analysis. In particular, the closer to the inside of the bubble, the weaker the signal was at 1160 cm^−1^, which appeared as a shoulder for positions 3–6. Moreover, the absorbance at 1022 cm^−1^ increased towards the inside.

Raman spectra collected through the section of bubbles also showed differences based on the depth of analysis (Figure 4c). Compared to the outer areas, the spectrum registered on the inner surface presented a strong OH band, a broad signal for the carbonyl group, and a fairly low intensity for the features of the nitro groups. The signal at 650 cm^−1^ resulted in being lower, and changes in the relative intensity of C-H stretching signals were observed.

Raman spectra were collected in depth also on a sample aged for 48 days at 30% RH to investigate the reason for the noted gain in fragility. The bulk material resulted in being depleted in the nitrate groups (lower intensity of the 1653 cm^−1^ band), while a new band at 445 cm^−1^ was registered below the external surface (point 3, Figure S7).

## 4. Discussion

The artificially aged samples were assessed to undergo significant changes both at the macro-scale and at the molecular level. They became more fragile and lost weight, and their appearance changed through yellowing and sometimes via the formation of cracks or the distortion into bubbles (Table 1). Spectroscopic analyses allowed the monitoring of the chemical transformations occurring simultaneously, so that some hypotheses on the origin of the “visible” alteration may be drawn.

**Figure 5:**
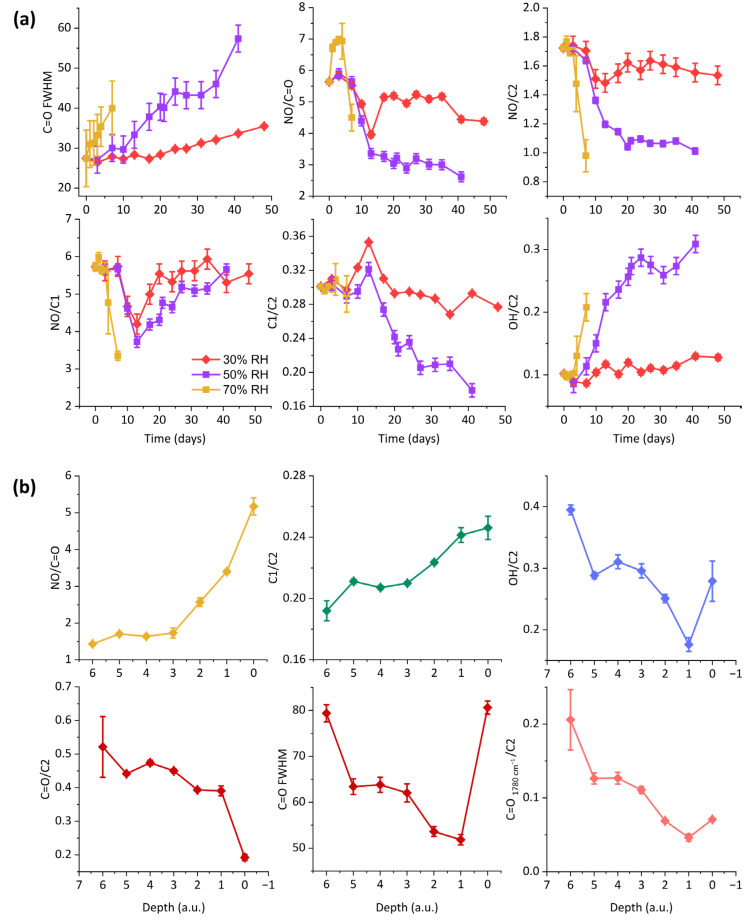
Intensity ratios calculated for representative ATR-FTIR signals on the surfaces of samples aged at 70 °C and 30%, 50%, and 70% RH over time (**a**) and through the section of bubbles formed after 7 days of ageing at 70 °C and 70% RH based on the arbitrary units assigned to depth (see Figure 4b) (**b**).

The parallel increase in absorption of the hydroxyl group and decrease in N-O-related signals assessed using infrared and Raman spectroscopy suggests the occurrence of denitration, which causes the release of nitrogen oxides and the substitution of the nitrate groups with OH functions [2,13,15,16,19,21,29,39]. Part of such hydroxyl groups were probably involved in oxidation reactions, giving rise to carbonyl-bearing species, as is likely to happen in celluloid through ageing [10,19,22,23]. Spectroscopic evidence of such a phenomenon was given by the broadening of the C=O band. By looking at the plots of Figure 5a, it is possible to notice that the mentioned phenomena occurred in all the ageing experiments, albeit to different extents. The relative intensity of the nitrate signal at 1635 cm^−1^ with respect to that of the carbonyl group at ca. 1730 cm^−1^ decreased in the three ageing tests. At 30% RH, this was rather limited; at 70% RH, it occurred between the fourth day and the end of the experiment; at 50% RH, a significant drop occurred between the seventh and thirteenth day of ageing. For a further characterisation of such evolution, the signals at 1160 and 1045 cm^−1^ were considered and are referred to as C1 and C2, respectively. Both signals are related to the C-O stretching vibrations, but their precise attribution is still rather controversial in the literature, to the extent that it is difficult to differentiate between the contribution of the glycosidic bond and the pyranoside groups [17,19,21,30,40,41]. It is interesting to note that the plots of the ratio NO/C2 resulted in almost being superimposable to those related to NO/C=O. Contrarily, a different scenario unveiled for the NO/C1 ratio, which first underwent a drop and subsequently increased in value. By considering that the ratio C1 over C2 was characterised by a decrease after 13 days of ageing, which is exactly the point of trend inversion for the NO/C1, it is possible to infer that the signal at 1160 cm^−1^ lost intensity with ageing, while that at 1045 cm^−1^ remained relatively constant. This assumed, one could interpret the drop in NO/C2 and NO/C=O as signals for a denitration process, taking place after 7 days of ageing at 70 °C and 30% or 50% RH. The parallel increase in OH/C2 starting after one week of ageing supports such a reading. In the case of the hydroxyl group, the slope did not reach a plateau after 13 days, but continued to rise. This is probably due to other hydrolysis reactions occurring on other sites of the polymer. Based on the trend inversion observed for the NO/C1 ratio after thirteen days, it is possible to hypothesise that the C1 signal is ascribed mainly to C-O of glycosidic bonds and that the reversal is related to the C-O-C bond cleavage. One could speculate that the degradation process occurs with an initial period where hydrolysis mainly induces denitration (with loss of NO signal and increase in pyranose C2 signal, and glycosidic bonds are minimally affected), followed by a phase where glycosidic bonds are attacked with depolymerisation. The breaking of glycosidic bonds and the consequent chain scission are thoroughly described as possible degradation phenomena for celluloid in the literature [16,19,21,42] and might be the reason for C1 decrease over time. The gradual increase in intensity of the band at 1022 cm^−1^ is also in accordance with such an interpretation, since, as suggested by Sinyayev et al. [40], this may be correlated with a modification in the degree of polymerisation of the material. Similarly, the Raman signal at 1151 cm^−1^ could be attributed to glycosidic bonds, and its decrease could be ascribedto chain disruption.

Besides denitration and chain scission, oxidation probably took place starting from the 13th day of ageing at 30 and 50% RH, as the carbonyl band started a gradual increase in width, which was especially marked for the higher-humidity scenario. It is no surprise that this happened just after the most pronounced denitration phase, as the released nitrogen oxides likely favoured the oxidation of celluloid, making the degradation process autocatalytic [2,13,15,16,21,39,43].

Some parallels among the described spectral modifications and the observed macroscopic changes may be drawn. By looking at the plot of mass loss, one can notice that the biggest decrease in mass for the ageing at 50% RH occurred between the 7th and the 20th day of ageing, which is the time interval where first a strong denitration took place and then the scission of glycosidic bonds started, probably causing the release of low molecular weight products [16,17,19,22]. For the ageing at 30% RH, the gradual and limited loss of weight is in accordance with the less pronounced denitration and decrease in C1 observed with the intensity ratio plots.

The chain scission process could be a reason for the embrittlement that affected the aged celluloid. Samples exposed to 50% RH presented cracks, probably due to the severity of this phenomenon, which was assessed using infrared spectroscopy. Fissures started to appear after 20 days of ageing, which correspond to the time needed for a large part of the glycosidic bonds to cleave. Furthermore, the decrease in intensity of the Raman signal at 650 cm^−1^ observed for the samples aged at 50% RH suggests that a partial loss of plasticiser took place. The occurrence of such phenomenon has been discussed in the literature by different authors [2,21] and could have contributed to the embrittlement and mass loss of the material.

The yellowing observed to occur through ageing may be associated with two phenomena. On the one hand, the NOx released through denitration may be partially trapped in the material and impart a yellowish hue [19,44]; on the other hand, chromophores may be included among the carbonyl-bearing species detected using infrared spectroscopy. Compared to the samples aged at 30% RH, those exposed to 50% RH resulted in a larger variation in b* value through ageing, suggesting that the higher the humidity, the greater the yellowing of the samples. The huge colour change registered at 50% RH on the sample used for contact angle measurements is in accordance: the sample had probably not completely dried after the contact angle analyses. Nevertheless, the value of b* decreased and that of a* increased for this sample, contrary to the general trend. Therefore, it is not clear whether the same phenomena led to this colour change and to the gradual hue shift registered throughout the ageing. Moreover, it must be noted that the major deviations from the values registered for the samples aged at 30% RH started after the 17th day of ageing. As crevices began forming right at that time in the case of the 50% RH ageing, it is likely that the greater yellowing registered by the colourimeter was enhanced by the higher scattering effect of the cracked samples.

Finally, the increase in roughness and the formation of bubbles and hollows observed using SEM and profilometry could have contributed to the promotion of the described degradation phenomena. By determining an increase in specific surface, they augmented the exposure to the humid surrounding environment already from the third day of ageing, just before the detection of the first spectroscopic changes. More analyses are necessary for the identification of the needle-shaped species formed after 20 days of ageing at 50% RH. Given the spectroscopic evidence for a strong denitration occurred at 50% RH before the 13th day of ageing, and considering the similarity to the micrographs reported in the literature, it could be hypothesised that such structures consist of microcrystalline cellulose [45,46].

The ageing at 70% RH deviated from the others, in that it was interrupted as soon as bubbles formed after one week. Infrared spectra collected on the samples surface suggested that, in such a timeframe, denitration and oxidation processes took place, while there was no evidence of glycosidic bond cleavage (C1/C2 ratio constant in this timeframe). As can be inferred from the plots of the intensity ratios, the decrease in nitrate groups and increase in hydroxyl functions started after 3 days, when samples began distorting. Subsequently, bubbles formed. Using µ-ATR-FTIR analyses, it was possible to note significant chemical differences across their section. The closer to the inside of the bubble, the more the material resulted denitrated, depolymerised, and oxidised. As can be seen in Figure 5b, the intensities of the nitrate group and of the C1 signal decreased towards the inner surface, while those of the hydroxyl group and of the carbonyl signals increased. More C=O-bearing species appeared to be present towards the inside of the bubble, as the carbonyl band resulted in becoming broader and more intense. Interesting to note is the discernability of the carbonyl shoulder at 1780 cm^−1^, which resulted in becoming higher with depth. Such a signal, corresponding to that registered with transmission FTIR spectroscopy, was also observed by Neves et al. after 225 h of artificial photo-oxidative ageing [17] and could be attributed to lactones or anhydrides species formed through oxidation [23,37].

Such results highlight the presence of a spatial gradient of alteration degree through the section of the samples aged at 70 °C and 70% RH. As the FTIR intensity ratios suggest, when exposed to such environment, celluloid went through a very fast denitration process. The released NOx gases could escape from the surface, whereas, in the bulk material, they probably accumulated due to the impossibility of prompt removal and caused the formation of bubbles. A highly oxidising environment likely developed in the inside of these, so that the degradation processes were catalysed in the inner surfaces. Other investigations are necessary for the proper study of the quantity and loss of plasticiser. Still, it may be hypothesised that camphor migrated from the bulk material towards the surface before being released to the environment. Thereby, the inner layers resulted in being depleted from the plasticiser, while the external ones had contents comparable to the unaged material.

## 5. Conclusions

The role of humidity in the high-temperature ageing of celluloid was examined in this study through the accelerated ageing of mock-up samples. When subjecting celluloid to 70 °C and three different levels of RH, it was possible to recognise a correlation between the degree of alteration and the moisture content in the ageing environment. At 30% RH, both the macroscopic and chemical changes were limited and hardly detectable, while at 70% RH, the samples were promptly altered and distorted by bubbles formation, so that the analyses were interrupted after one week. The test conducted at 50% RH caused significant but slower modifications on the samples, allowing the recognition of the main processes of degradation. The combination of infrared and Raman spectroscopy with microscopy, mass measurements, profilometry, and colourimetry gave the means to reveal meaningful parallelisms between the chemical and macroscopic modifications. Evidence was given for the interlacement of denitration, chain scission, and oxidation phenomena, as well as the assessed embrittlement and morphological and colour changes. Given the different outcomes for the three ageing experiments, humidity played a substantial role in the degradation of celluloid. Still, its impact was probably intertwined with the effect of the high temperature used in the tests. Further experiments at 50 and 25 °C are planned and will aim to study the synergetic roles of RH and T in the framework of more comprehensive and systematic research on the ageing of celluloid. A verification of the results obtained through the accelerated ageing tests will be implemented in future works through a non-invasive investigation on naturally aged celluloid objects.

## Figures and Tables

**Table 1:** Summary of the physical and macroscopic changes occurring on celluloid samples aged at 70 °C and 30%, 50%, and 70% RH. The number of days after which the macroscopic changes occurred is reported in brackets. The mean roughness measured through profilometry is indicated as Ra.

Ageing RH (%)	Duration (Days)	Mass Loss (%)	Macroscopic Changes	Ra Max (µm)
30	48	10	Embrittlement (27)	0.15
50	41	32	Cracking (20)	0.14
70	7	17	Bubbles (7)	1.95

## Data Availability

The data presented in this study are available on request from the corresponding author.

## Supplementary Materials

The following supporting information can be downloaded at: https://www.mdpi.com/article/10.3390/polym17121648/s1, Figure S1: Systems for the ageing experiments at T ≥ 30 °C are shown in (**a**). Desiccators where the desired RH level is attained with a defined saturated salt solution, are put in the oven. In (**b**) part of the data collected while monitoring the behaviour of the system is displayed. The levels of T and RH% registered in the frame of about 8 h for systems having a NaCl saturated solution and being exposed to ambient temperature, 30 or 50 °C are plotted against time. Perturbations to the systems are indicated with p1 and p2 with colours related to the different temperatures of the systems; Figure S2: The contact angle average values registered on samples aged at 70 °C and with 30, 50 and 70% RH are plotted versus the time of ageing: all the ageing experiments are considered in (**a**), showing the measures registered in the first week, while the values collected for the two long-term ageing tests (30 and 50% RH) are reported in (**b**). Drop of distilled water for contact angle measurements on the surface of an artificially aged celluloid sample (70 °C, 50% RH, 24 days) at the first contact with the surface (0 s) and after 5 and 20 s (**c**). The measured c.a. values are 77.86, 66.37 and 49.07 degrees, respectively; Figure S3: a* and b* colourimetric values (means of five acquisitions) registered for samples aged at 70 °C are plotted versus the time of ageing, respectively in (**a**) and (**b**). The values collected in the 41st day of 50% RH ageing are indicated with a * and are not linked to the foregoing points, as they refer to the sample used for contact angle measurements; Figure S4: SEM micrographs of pristine celluloid (**a**,**f**) and samples aged at 70 °C and 30% RH for (**b**,**g**) 3, (**c**,**h**) 7, (**d**,**i**) 13 and (**e**,**j**) 48 days. Scales are common for images (**a**–**e**) and for (**f**–**j**); Figure S5: SEM micrographs of pristine celluloid (**a**,**f**) and samples aged at 70 °C and 50% RH for (**b**,**g**) 3, (**c**,**h**) 13, (**d**,**i**) 20 and (**e**,**j**) 24 days. Scales are common for images (**a**–**e**) and for (**f**–**j**); Figure S6: SEM micrographs of celluloid samples aged at 70 °C and 50% RH for 41 days (**a**–**d**) and 70% RH for 7 days (**e**–**h**). For the ageing at lower RH%, crystals formed on the surface are displayed in (**a**,**c**,**d**) (in the latter image, a crystal flake on a corner of a sample fragment is shown), while the surface of a freshly cut section of the sample is reported in (**b**). For the ageing at 70% RH, images (**e**,**f**) display the surface and micrographs (**g**,**h**) the side of the analysed sample; Table S1: Infrared and Raman bands assignment for celluloid; Figure S7: Transmission FTIR spectra registered on celluloid samples before and after ageing at 70 °C and 30%RH for 48 days, 50% RH for 41 days and 70% RH for 7 days (the wavenumbers of the discussed signals are reported in black) (**a**) and Raman spectra collected at different depths in sections of celluloid samples aged at 70 °C and 30% RH for 48 days (**c**). The approximate position of Raman spectra acquisition in the sample section is shown in (**b**).
