# Supplementary material for: The Impact of Humidity in the Thermal Ageing of Celluloid: An Inter-Scale Investigation
Source: Polymers (Basel). 2025 Jun 13;17(12):1648. doi: 10.3390/polym17121648 (PMC12197267; doi:10.3390/polym17121648)
Supplement: Supplementary file 1 [file polymers-17-01648-s001.zip › polymers-3680716-supplementary.pdf]

Supplementary material

1. Set up of the ageing protocol

The ageing protocol was tested before the experiments on the samples. The temperature of the oven was checked to be homogeneous in the whole volume of its chamber. To monitor the RH levels of the systems placed either inside or outside the oven (set to 50 °C and 30 °C), Oregon Scientific Data Loggers were placed inside the desiccators. For the tests, NaCl and $MgCl_2$ were employed: in both cases, several hours were necessary for reaching stable conditions, and, regardless of temperature, letting the system stabilise overnight turned out to be preferable. The systems were also subjected to perturbations, consisting in a simulation of sample withdrawal for the analyses: desiccators in the oven were taken out and opened for a few seconds before being reset in their initial condition. Such procedure caused non-negligible changes to the controlled environment inside the desiccators, as the RH level was registered to fall of about 10% in systems at 30 or 50 °C and of 3% in the system placed in the laboratory environment, at room temperature (between 20 and 25 °C). In order for the relative humidity to reach again the initial level, more than 3 hours were necessary for the systems in the oven, whereas one hour and a half was sufficient for that at room T. Fundamental were the performed tests to assess that the systems placed in the oven require a non-negligible time interval to reach stability. This considered, it was decided to stabilise overnight the systems prior to the beginning of the ageing experiments, and to perform analyses at most once a day, keeping the samples outside their ageing environment for the shortest time possible. For the ageing tests on the samples with RH of 70%, 50% and 30%, saturated salt solutions were prepared in about 70 mL of deionised water, with ca. 50 g of NaCl, 100 g of $Mg(NO_3)_2$ and 80 g of $MgCl_2$ respectively. The chosen amounts were largely exceeding the solubility limit for the three salts (respectively, 36 g/100mL, 70 g/100 mL and 55 g/mL), resulting in saturated solutions with sediment deposited on the ground.

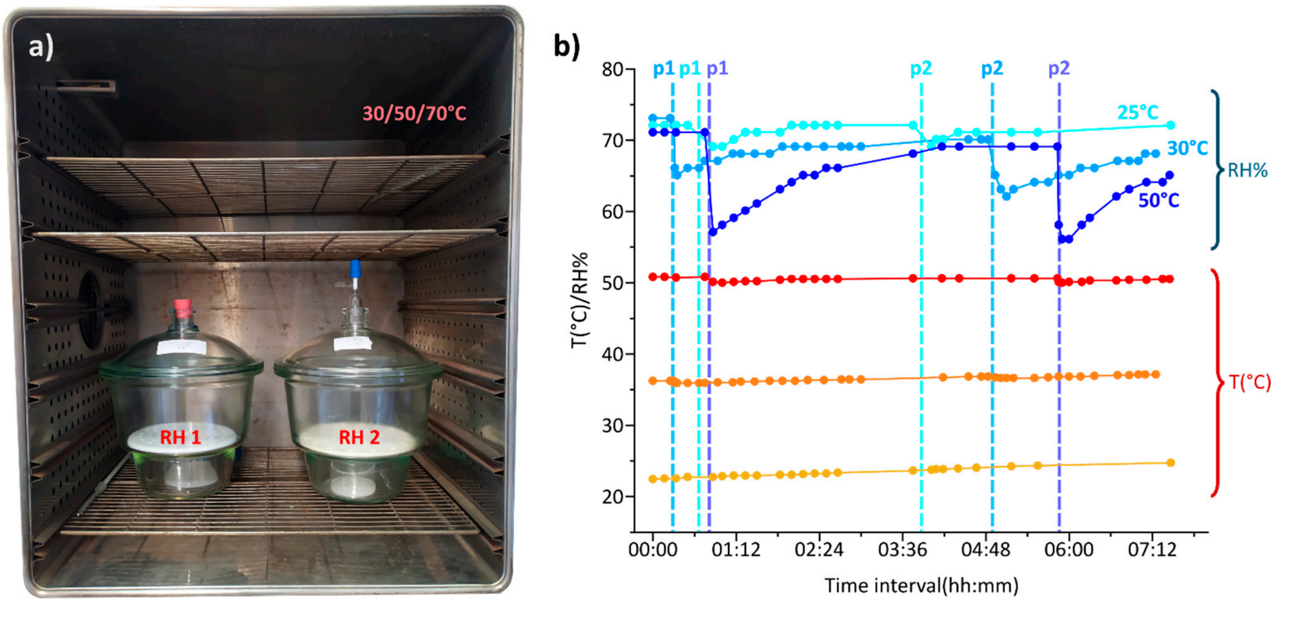

Figure S1. Systems for the ageing experiments at T≥30 °C are shown in **(a)**. Desiccators where the desired RH level is attained with a defined saturated salt solution, are put in the oven. In **(b)** part of the data collected while monitoring the behaviour of the system is displayed. The levels of T and RH% registered in the frame of about 8 hours for systems having a NaCl saturated solution and being exposed to ambient temperature, 30 or 50 °C are plotted against time. Perturbations to the systems are indicated with p1 and p2 with colours related to the different temperatures of the systems.

2. Contact angle measurements

In Figure S2 the mean values of contact angle (c.a.) measures for the aged samples are reported. By looking at the plot in (a), it is possible to compare the results obtained for the first week of ageing in the experiments performed at the same temperature (70 °C), but at different humidity levels. The trend can be considered equal for the ageing performed at 30 and 50% RH and indicated a slight increase in hydrophobicity of the surface. In the case of 70% RH, considerably lower values were registered after one week. The longer experiments performed at lower humidity (b) showed that, in about 40 days, the 30% RH ageing led to an overall decrease in wettability, whereas that at 50% RH had the opposite effect. Starting from the 20th day of ageing, not only were the c.a. measures low at the first contact of the drop with the surface, but they also decreased very rapidly in the following 20 seconds. As can be seen in the example of Figure S2 (c), drops expanded progressively on the aged surface until reaching a quite stable c.a., ranging from about 30 to 70°. Water was likely absorbed by the celluloid sheets, since a decrease in drop volume was registered parallel to the reduction of contact angle.

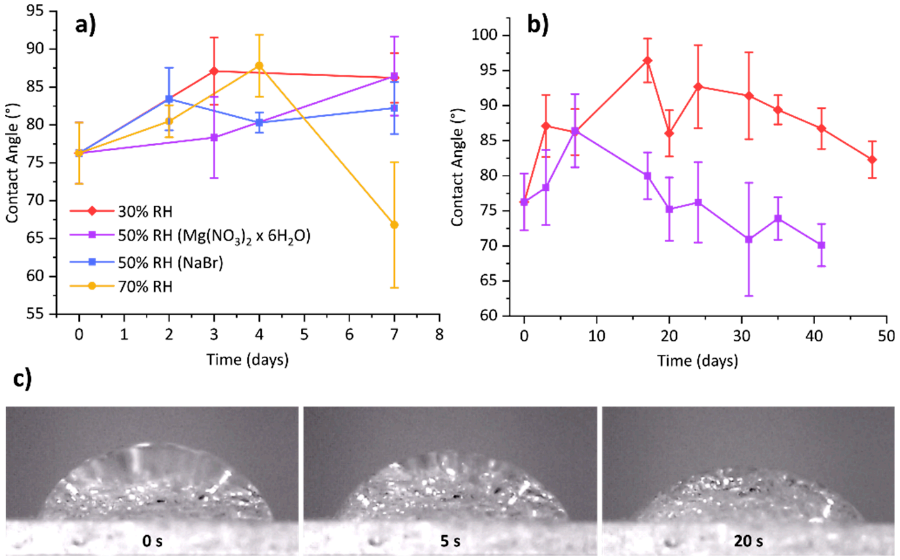

Figure S2. The contact angle average values registered on samples aged at 70 °C and with 30, 50 and 70% RH are plotted versus the time of ageing: all the ageing experiments are considered in **(a)**, showing the measures registered in the first week, while the values collected for the two long-term ageing tests (30 and 50% RH) are reported in **(b)**. Drop of distilled water for contact angle measurements on the surface of an artificially aged celluloid sample (70 °C, 50% RH, 24 days) at the first contact with the surface (0 seconds) and after 5 and 20 seconds **(c)**. The measured c.a. values are 77.86, 66.37 and 49.07 degrees, respectively.

3. Colourimetry

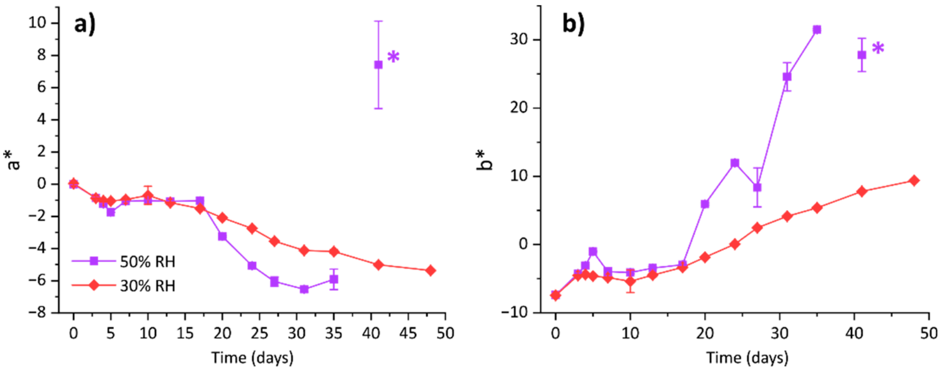

Figure S3. a* and b* colourimetric values (means of five acquisitions) registered for samples aged at 70 °C are plotted versus the time of ageing, respectively in **(a)** and **(b)**. The values collected in the 41st day of 50% RH ageing are indicated with a * and are not linked to the foregoing points, as they refer to the sample used for contact angle measurements.

4. Scanning Electron Microscopy (SEM)

For an overview on the morphological changes occurring on the sample surface, a selection of SEM images acquired for both the unaged material and samples aged at 70 °C and 30% or 50% RH is reported in Figure S4 and Figure S5. Further details on the crystalline species formed on the samples aged at 70 °C and 50% RH are shown in Figure S6, together with SEM micrographs acquired on the surface and the side of samples aged for 7 days at 70 °C and 70% RH.

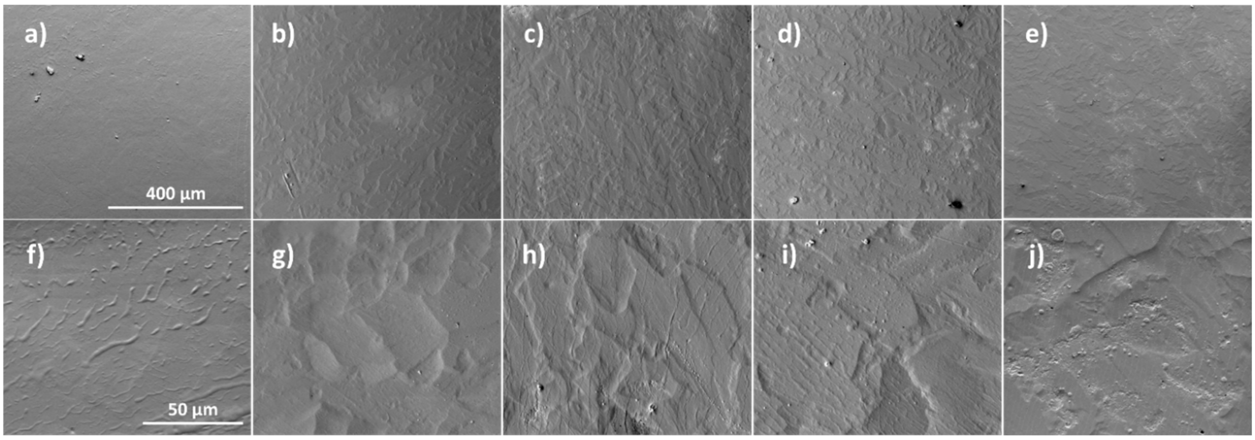

Figure S4. SEM micrographs of pristine celluloid **(a,f)** and samples aged at 70 °C and 30% RH for **(b,g)** 3, **(c,h)** 7, **(d,i)** 13 and **(e,j)** 48 days. Scales are common for images **(a-e)** and for **(f-j)**.

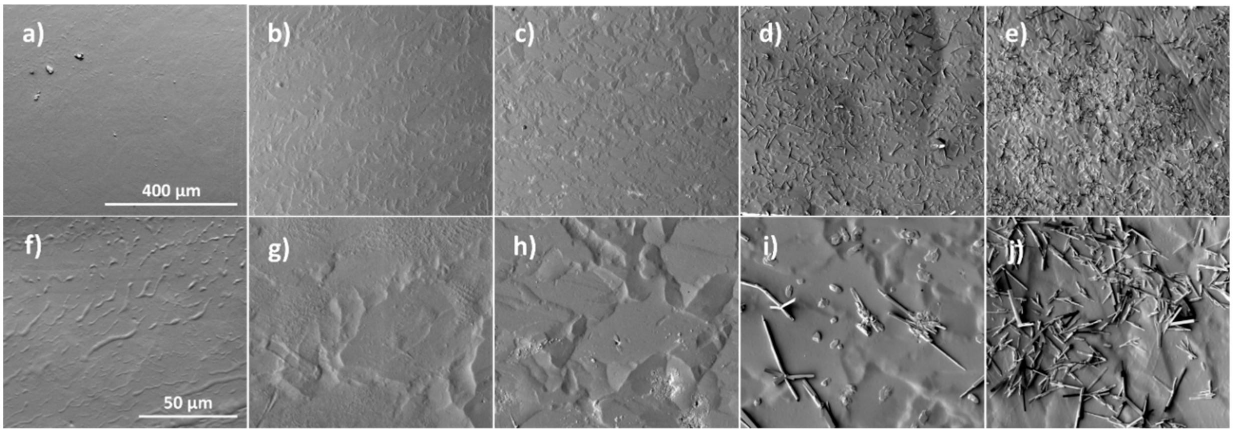

Figure S5. SEM micrographs of pristine celluloid **(a,f)** and samples aged at 70 °C and 50% RH for **(b,g)** 3, **(c,h)** 13, **(d,i)** 20 and **(e,j)** 24 days. Scales are common for images **(a-e)** and for **(f-j)**.

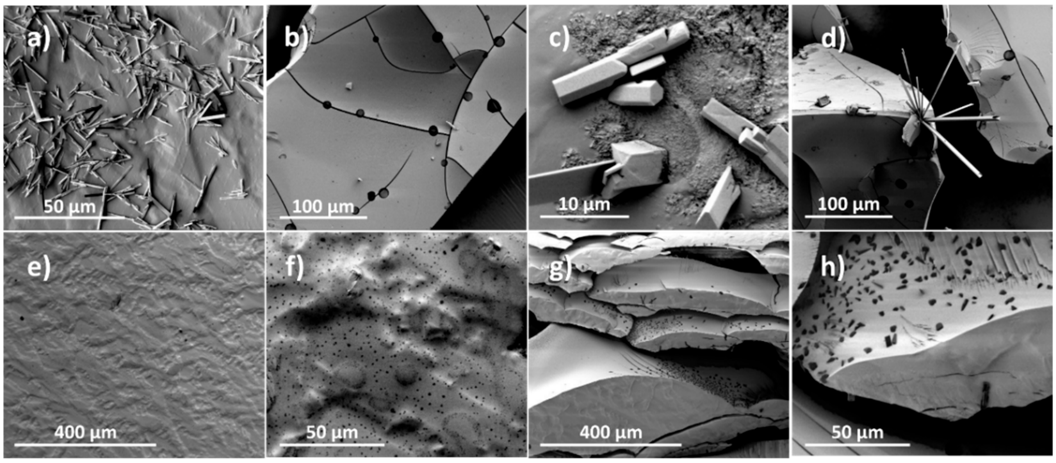

Figure S6. SEM micrographs of celluloid samples aged at 70 °C and 50% RH for 41 days **(a-d)** and 70% RH for 7 days **(e-h).** For the ageing at lower RH%, crystals formed on the surface are displayed in **(a,c,d)** (in the latter image, a crystal flake on a corner of a sample fragment is shown), while the surface of a freshly cut section of the sample is reported in **(b)**. For the ageing at 70% RH, images **(e,f)** display the surface and micrographs **(g, h)** the side of the analysed sample.

5. Infrared and Raman spectroscopy

Table S1. Infrared and Raman bands assignment for celluloid.

INFRARED	ASSIGNMENT	RAMAN	ASSIGNMENT
3430 *b w*	ν O-H [1–5]	3530 *b w*	ν O-H [6]
2970-2850 *w*	ν CH and ν CH_2 [1,4,5,7–10]	2970 *vs*, 2930 *vs*,<br>2905 *sh*, 2876 *sh*	ν CH [1,4,6]
1728 *m*	ν C=O of camphor [1,5,8,10–12]	1730 *m*	ν C=O of camphor [4,13,14]
1636 *vs*	$ν_a$ O-NO_2 [1–5,8,10,12,15]	1657 *m*	$ν_a$ NO_2 [1,4,13,14]
1454 *w*	δ CH_2 [1–5]	1450 *m*	δ CH_2 camphor [1,4,14]
1428 *w*	δ CH_2 [5,8]<br>δ C-OH [1,4,16]		
1417 *w*	δ CH_2 of camphor [5,17]	1417 *m*	δ C-OH [1]
1390 *sh*	$δ_s$ CH_3 [5,17]	1373 *m*	δ CH [1,4,13,14]
1375 *m*	δ C-H of CN [1–5,7,8,16]<br>$δ_s$ CH3 and ν C-C of camphor [17]		
1325 *w*	ω CH_2, ν C-C, $δ_s$ CH_3 at C1 of camphor [5,17]	1326 *w*	ω CH_2[1]
1273 *vs*	$ν_s$ NO_2 of CN [1–5,7,8,10–12,15,16]<br>ω CH_2 , ν CC and ring deformation of camphor [17]	1285 *s*	$ν_s$ NO_2 [1,4,13,14]
		1248 *w*<br>1221 *w*	
1207 *w*<br>1160 *m*<br>1115 *sh*<br>1052 *s*<br>999-1022 *s*	ν COC [1–5,8,10,11,15,16]	1201 *vw*<br>1154 *m*<br>1126 *m*<br>1097 *m*<br>1081 *w*	ν COC [1,4,6]
		1024 *w*	$ν_s$ NO_3^- [4]
		1013 *w*	ν CO [1]
		987 *w*	str C-O-C [4,13,14]
918-47 *w*	$δ_s$ CH [1,5]	950 *m*	$ν_s$ N-(OH) [4]
828 *vs*	ν NO [1,5,10–12,15]	926 *sh*	$ν_s$ N-(OH) [4]
		916 *m*	$δ_s$ CH [1]
		863 *s*	$δ_s$ CH of camphor [13]
		854 *s*	ν NO [1,14]
750 *m*	δ O-NO_2 [5,8,12,18]<br>$γ_w$ NO_2 [1,4,16]	753 *w*	ω NO_2[1]
722 *w*		710 *w*	δ O-N-O [14]<br>camphor [14]
695 *sh*	δ NO_2 [1–5,7,8,12]	699-700 *sh*	δ NO_2 [1,4,13,14]
677 *m*	τ NO_2 [18,19]		
650 *w*	ν NO [18]	650-2 *vs*	ring deformation of camphor [4,13,14]
626 *m*	$γ_r$ NO_2 [18,19]	625-9 *vw*	$γ_r$ NO_2 [1,4,14]
575 *w*	δ C-C-O [18]	610 *w*	
553 *w*	δ C-C-O [18]	557 *m*	camphor [13]
543 *vw*	δ O-N-O [18,19]	523 *w*	
522 *w*	degree of substitution (DS) related [18]	475 *w*	
450-420 *w*	δ O-N-O [18,19]	396 *w*	
		303 *vw*<br>263 *w*<br>246 *w*<br>123 *s*	

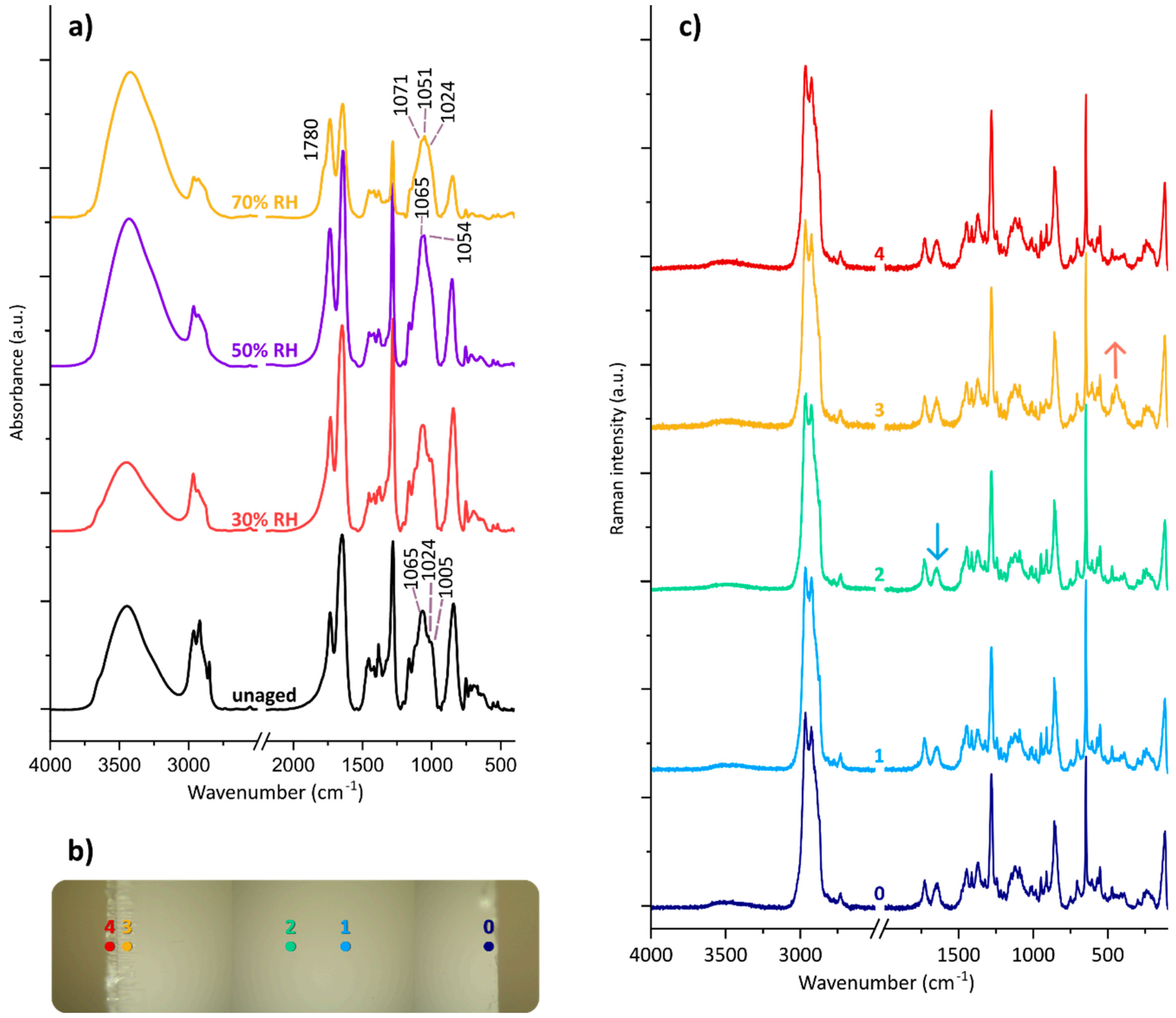

Figure S7. Transmission FTIR spectra registered on celluloid samples before and after ageing at 70 °C and 30%RH for 48 days, 50% RH for 41 days and 70% RH for 7 days (the wavenumbers of the discussed signals are reported in black) **(a)** and Raman spectra collected at different depths in sections of celluloid samples aged at 70 °C and 30% RH for 48 days **(c)**. The approximate position of Raman spectra acquisition in the sample section is shown in **(b)**.

References

1. Moore, D.S.; McGrane, S.D. Comparative Infrared and Raman Spectroscopy of Energetic Polymers. *J. Mol. Struct.* **2003**, *661–662*, 561–566, doi:10.1016/S0022-2860(03)00522-2.
2. Bussiere, P.-O.; Gardette, J.-L.; Therias, S. Photodegradation of Celluloid Used in Museum Artifacts. *Polym. Degrad. Stab.* **2014**, *107*, 246–254, doi:10.1016/j.polymdegradstab.2014.02.022.
3. Berthumeyrie, S.; Collin, S.; Bussiere, P.-O.; Therias, S. Photooxidation of Cellulose Nitrate: New Insights into Degradation Mechanisms. *J. Hazard. Mater.* **2014**, *272*, 137–147, doi:10.1016/j.jhazmat.2014.02.039.
4. Neves, A.; Angelin, E.M.; Roldão, É.; Melo, M.J. New Insights into the Degradation Mechanism of Cellulose Nitrate in Cinematographic Films by Raman Microscopy. *J. Raman Spectrosc.* **2019**, *50*, 202–212, doi:10.1002/jrs.5464.
5. Chavez Lozano, M.V.; Elsässer, C.; Angelin, E.M.; Pamplona, M. Shedding Light on Degradation Gradients in Celluloid: An ATR-FTIR Study of Artificially and Naturally Aged Specimens. *Polymers* **2023**, *15*, 522, doi:10.3390/polym15030522.
6. Socrates, G. *Infrared and Raman Characteristic Group Frequencies: Tables and Charts*; 3. ed., repr. as paperback.; Wiley: Chichester, 2010; ISBN 978-0-470-09307-8.
7. Jutier, J.-J.; Harrison, Y.; Premont, S.; Prud'homme, R.E. A Nonisothermal Fourier Transform Infrared Degradation Study of Nitrocelluloses Derived from Wood and Cotton. *J. Appl. Polym. Sci.* **1987**, *33*, 1359–1375, doi:10.1002/app.1987.070330424.
8. Quye, A.; Littlejohn, D.; Pethrick, R.A.; Stewart, R.A. Investigation of Inherent Degradation in Cellulose Nitrate Museum Artefacts. *Polym. Degrad. Stab.* **2011**, *96*, 1369–1376, doi:10.1016/j.polymdegradstab.2011.03.009.
9. Mitchell, G.; France, F.; Nordon, A.; Tang, P.L.; Gibson, L.T. Assessment of Historical Polymers Using Attenuated Total Reflectance-Fourier Transform Infra-Red Spectroscopy with Principal Component Analysis. *Herit. Sci.* **2013**, *1*, 28, doi:10.1186/2050-7445-1-28.
10. Nunes, S.; Ramacciotti, F.; Neves, A.; Angelin, E.M.; Ramos, A.M.; Roldão, É.; Wallaszkovits, N.; Armijo, A.A.; Melo, M.J. A Diagnostic Tool for Assessing the Conservation Condition of Cellulose Nitrate and Acetate in Heritage Collections: Quantifying the Degree of Substitution by Infrared Spectroscopy. *Herit. Sci.* **2020**, *8*, 33, doi:10.1186/s40494-020-00373-4.
11. Pereira, A.; Candeias, A.; Cardoso, A.; Rodrigues, D.; Vandenabeele, P.; Caldeira, A.T. Non-Invasive Methodology for the Identification of Plastic Pieces in Museum Environment — a Novel Approach. *Microchem. J.* **2016**, *124*, 846–855, doi:10.1016/j.microc.2015.07.027.
12. Izzo, F.C.; Carrieri, A.; Bartolozzi, G.; Keulen, H.V.; Lorenzon, I.; Balliana, E.; Cucci, C.; Grazzi, F.; Picollo, M. Elucidating the Composition and the State of Conservation of Nitrocellulose-Based Animation Cells by Means of Non-Invasive and Micro-Destructive Techniques. *J. Cult. Herit.* **2019**, *35*, 254–262, doi:10.1016/j.culher.2018.09.010.
13. Paris, C.; Coupry, C. Fourier Transform Raman Spectroscopic Study of the First Cellulose-Based Artificial Materials in Heritage. *J. Raman Spectrosc.* **2005**, *36*, 77–82, doi:10.1002/jrs.1288.
14. Neves, A.; Friedel, R.; Callapez, M.E.; Swank, S.D. Safeguarding Our Dentistry Heritage: A Study of the History and Conservation of Nineteenth–Twentieth Century Dentures. *Herit. Sci.* **2023**, *11*, 142, doi:10.1186/s40494-023-00989-2.
15. Silverstein, R.M.; Webster, F.X.; Kiemle, D.J. *Spectrometric Identification of Organic Compounds*; 7th ed.; John Wiley & Sons: Hoboken, NJ, 2005; ISBN 978-0-471-39362-7.
16. Castro, K.; De Vallejuelo, S.F.; Astondoa, I.; Goñi, F.M.; Madariaga, J.M. Analysis of Confiscated Fireworks Using Raman Spectroscopy Assisted with SEM-EDS and FTIR. *J. Raman Spectrosc.* **2011**, *42*, 2000–2005, doi:10.1002/jrs.2946.
17. Abbate, S.; Burgi, L.F.; Gangemi, F.; Gangemi, R.; Lebon, F.; Longhi, G.; Pultz, V.M.; Lightner, D.A. Comparative Analysis of IR and Vibrational Circular Dichroism Spectra for a Series of Camphor-Related Molecules. *J. Phys. Chem. A* **2009**, *113*, 11390–11405, doi:10.1021/jp905644d.
18. Mukhamadeeva, R.M.; Zhbankov, R.G.; Sopin, V.F.; Marchenko, G.N. Low-Frequency IR Spectroscopy in the Study of the Structures of Cellulose and Cellulose Nitrates. *Russ. Chem. Rev.* **1993**, *62*, 323–336, doi:10.1070/RC1993v062n04ABEH000020.
19. Wei, R.; Huang, S.; Wang, Z.; Wang, C.; Zhou, T.; He, J.; Yuen, R.; Wang, J. Effect of Plasticizer Dibutyl Phthalate on the Thermal Decomposition of Nitrocellulose. *J. Therm. Anal. Calorim.* **2018**, *134*, 953–969, doi:10.1007/s10973-018-7521-3.
